# Goal-Directed Resuscitation Aiming Cardiac Index Masks Residual Hypovolemia: An Animal Experiment

**DOI:** 10.1155/2015/160979

**Published:** 2015-10-12

**Authors:** Krisztián Tánczos, Márton Németh, Domonkos Trásy, Ildikó László, Péter Palágyi, Zsolt Szabó, Gabriella Varga, József Kaszaki

**Affiliations:** ^1^Department of Anaesthesiology and Intensive Therapy, Faculty of Medicine, University of Szeged, 6 Semmelweis Street, Szeged 6725, Hungary; ^2^Institute of Surgical Research, University of Szeged, 6 Semmelweis Street, Szeged 6725, Hungary

## Abstract

The aim of this study was to compare stroke volume (SVI) to cardiac index (CI) guided resuscitation in a bleeding-resuscitation experiment. Twenty six pigs were randomized and bled in both groups till baseline SVI (*T*_bsl_) dropped by 50% (*T*_0_), followed by resuscitation with crystalloid solution until initial SVI or CI was reached (*T*_4_). Similar amount of blood was shed but animals received significantly less fluid in the CI-group as in the SVI-group: median = 900 (interquartile range: 850–1780) versus 1965 (1584–2165) mL, *p* = 0.02, respectively. In the SVI-group all variables returned to their baseline values, but in the CI-group animals remained underresuscitated as indicated by SVI, heart rate (HR) and stroke volume variation (SVV), and central venous oxygen saturation (S_cv_O_2_) at *T*_4_ as compared to *T*_bsl_: SVI = 23.8 ± 5.9 versus 31.4 ± 4.7 mL, HR: 117 ± 35 versus 89 ± 11/min SVV: 17.4 ± 7.6 versus 11.5 ± 5.3%, and S_cv_O_2_: 64.1 ± 11.6 versus 79.2 ± 8.1%, *p* < 0.05, respectively. Our results indicate that CI-based goal-directed resuscitation may result in residual hypovolaemia, as bleeding caused stress induced tachycardia “normalizes” CI, without restoring adequate SVI. As the SVI-guided approach normalized most hemodynamic variables, we recommend using SVI instead of CI as the primary goal of resuscitation during acute bleeding.

## 1. Introduction

Acute bleeding due to trauma, surgery, or gastrointestinal disorders is a life threatening condition requiring immediate and adequate interventions, of which intravenous fluid therapy is regarded as the first step of resuscitation. Although lifesaving at the time, inadequate fluid resuscitation can lead to hypo- or hyper-perfusion causing the development of multiorgan disorders at a later stage, which then severely affects the outcome of these patients [1, 2]. Therefore, the use of early and efficient therapeutic strategies able to detect and to treat the imbalance between oxygen delivery and consumption is of particular importance in critically ill patients, which has been recognized for decades [3].

Traditional endpoints of resuscitation, such as heart rate, blood pressure, mental status, and urine output can be useful in the initial identification of inadequate perfusion but are limited in their ability to identify ongoing, compensated shock [4]. More detailed assessment of global macrohemodynamic indices such as cardiac output and derived variables, measures of oxygen debt, may be necessary to guide treatment [5, 6].

Cardiac output calculated from thermodilution or pulse contour analysis is the most often used end-point during goal-directed therapy [7, 8]. However, there is no consensus on the best or universally accepted parameter as resuscitation target. In a recent animal experiment we described changes in central venous oxygen saturation (S_cv_O_2_) and venous-to-arterial carbon dioxide gap (dCO_2_) during an experimental stroke volume index- (SVI-) guided bleeding and fluid resuscitation model on porcine. We found that dCO_2_ may be a useful hemodynamic endpoint of resuscitation, while S_cv_O_2_ is not strictly a hemodynamic parameter, but rather an indicator of the balance between oxygen delivery and consumption [9]. However, we also noticed that normalizing stroke volume index resulted in higher cardiac index (CI) by the end of resuscitation as compared with baseline, possibly because of the bleeding-induced tachycardia. Hence we hypothesized that normalizing cardiac output only may mask ongoing hypovolemia due to increased heart rate caused by sympathetic response and may result in inadequate fluid resuscitation. Therefore, the objective of the current study was to compare SVI as primary target of fluid resuscitation to CI-based treatment in a bleeding-resuscitation animal model.

## 2. Materials and Methods

The experiments were performed on the EU Directive 2010/63/EU on the protection of animals used for experimental and other scientific purposes and carried out in strict adherence to the NIH guidelines for the use of experimental animals. The study was approved by the National Scientific Ethical Committee on Animal Experimentation (National Competent Authority), with the license number V./142/2013.

### 2.1. Animals and Instrumentation

Vietnamese mini-pigs (*n* = 27) underwent a 12-hour fasting preoperatively but with free access to water. Anesthesia was induced by intramuscular injection of a mixture of ketamine (20 mg/kg) and xylazine (2 mg/kg) and maintained with a continuous intravenous infusion of propofol (6 mg/kg/hr iv.), while analgesia was performed with nalbuphine (0.1 mg/kg). The animals' trachea was intubated and the lungs were ventilated mechanically with Dräger Evita XL (Dräger, Lübeck, Germany). The tidal volume was set at 10 mL/kg, and the respiratory rate was adjusted to maintain the end-tidal carbon dioxide and partial pressure of arterial carbon dioxide in the range of 35–45 mmHg. The adequacy of the depth of anesthesia was assessed by monitoring the jaw tone. After induction of anesthesia, the right jugular vein, the left carotid artery, and the right femoral artery were dissected and catheterized using aseptic technique. For invasive hemodynamic monitoring, a transpulmonary thermodilution catheter (PiCCO, PULSION Medical Systems SE, Munich, Germany) was placed in the right femoral artery. Central venous catheter was inserted via the right jugular vein and was positioned by the guidance of intracavital ECG. During the bleeding phase blood was drained from a sheat inserted in the left carotid artery. Animals were kept warm (37 ± 1°C) by an external warming device.

### 2.2. Hemodynamic Monitoring and Blood Gas Sampling

Cardiac output (CO), global end-diastolic volume index (GEDI), stroke volume (SV), cardiac function index (CFI), index of left ventricular contractility (dPmax), SV variation (SVV), pulse pressure variation (PPV), heart rate (HR), and mean arterial pressure (MAP) were measured by transpulmonary thermodilution and pulse contour analysis at baseline and at the end of each interval. All hemodynamic parameters were indexed for body surface area or bodyweight. Central venous catheter was used for the injection of cold saline boluses for the thermodilution measurements. The average of three measurements following 10 mL bolus injections of ice-cold 0.9% saline was recorded. Central venous pressure (CVP) was measured via central venous catheter at the same times as the other hemodynamic variables.

For blood gas measurements the right femoral artery served as the site for arterial blood gas sampling and the central venous line was used for taking central venous blood gas samples, which were analyzed by cooximetry (Cobas b 221, Roche Ltd., Basel, Switzerland) simultaneously at baseline and at the end of each step. From these parameters the following variables were calculated:

(1) Delivery  of  oxygen  DO2=CI∗Hb∗1.34∗SaO2+0.003∗PaO2,Oxygen  consumption  VO2=CI∗CaO2−Hb∗1.34∗ScvO2+0.003∗PcvO2,Oxygen  extraction=VO2DO2.

### 2.3. Experimental Protocol

**Figure 1:**
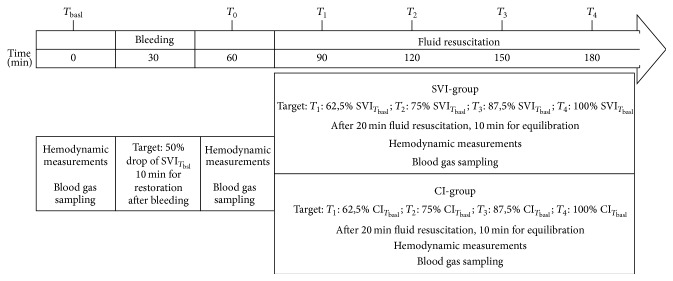
Flow chart. Schematic diagram illustrating the flowchart of the experimental protocol. After baseline measurement, animals were bled until the stroke volume index (SVI) decreased by 50% (*T*_0_); then measurements were repeated and randomized into two group. In the SVI-group the difference of the SVI_*T*_bsl__ − SVI_*T*_0__ was divided into four equal target values (*T*_1–4_), and fluid resuscitated to reach the initial SVI by *T*_4_. In the CI-group the difference of the CI_*T*_bsl__ − CI_*T*_0__ was divided into 4 target values and then the animals were resuscitated in 4 steps in order to reach the CI_*T*_bsl__ by *T*_4_.

The flowchart of the experiment is summarized in Figure 1. After the instrumentation, animals were allowed to rest for 30 minutes after which baseline (*T*_bsl_) hemodynamic, microcirculatory measurements, blood gas analyses, including lactate measurements, and laboratory testing were performed. After these measurements, blood was drained until the stroke volume index dropped by 50% of its baseline value (*T*_0_); then measurements were repeated. At this point the animals were randomized into two groups. In the SVI-group the difference of the SVI_*T*_bsl__ − SVI_*T*_0__ was divided into four equal target values, which was aimed to reach in 4 steps during fluid resuscitation (*T*_1–4_) to reach the initial SVI by *T*_4_. While in the CI-group the difference of the CI_*T*_bsl__ − CI_*T*_0__ was divided into 4 target values and then the animals were resuscitated in 4 steps in order to reach the CI_*T*_bsl__ by *T*_4_. Fluid replacement was carried out with boluses of 200 mL of balanced crystalloid Ringerfundin (B. Braun AG., Melsungen, Germany) over 10 minutes, till the target SVI or CI value was reached. After reaching each step, 20 minutes was allowed for equilibrium; then hemodynamic and blood gas parameters were measured. At the end of the experiment the animals were euthanized with sodium pentobarbital.

### 2.4. Data Analysis and Statistics

Data are presented as mean ± standard deviations unless indicated otherwise. For testing normal distribution the Kolmogorov-Smirnov test was used. Changes in all parameters throughout the experiment were tested by two-way repeated measures analysis of variance (RM ANOVA) and for the post hoc test Bonferroni test was used. For pairwise comparisons Pearson's correlation was used. The primary end point of the study was the normalization of SVV, as the one of the best indicators of hypo-, normovolemia in mechanically ventilated subjects [10]. Based on the results of our previous animal experiment [9] SVV was found to be 12.2 ± 4.3% by the end of resuscitation. Considering that CI-based resuscitation remains inadequate, we regarded a clinically significant difference of 4% (i.e., 12% in the SVI-group and 16% in the CI-group). In order the study to have 80% power to show a difference between the two groups if *α* < 0.05, the required sample size is a minimum of 20 animals (10 in each group). For statistical analysis SPSS version 20.0 for Windows (SPSS, Chicago, IL) was used and *p* < 0.05 was considered statistically significant.

## 3. Results

All animals survived the experiment, apart from one (CI-group), which had sudden cardiac arrest after induction of anesthesia for unknown reasons. Therefore, the results of 14 animals in the SVI-group and 12 animals in the CI-group were analyzed. Demographics and fluid management data are summarized in Table 1. Animals were of similar weight in both groups. For a 50% decrease of SVI similar blood had to be drained in the two groups. During resuscitation animals in the SVI-group required more fluid in total, and taking into account the volume of crystalloid required to replace a unit of 10 mL blood loss, animals in the SVI-group also received significantly more fluid (Table 1).

### 3.1. Macrohemodynamics

Hemodynamic parameters were similar at *T*_bsl_ and goals of 50% reduction in SVI were reached by *T*_0_ in both groups (Table 2). In the SVI-group SVI returned to its baseline value by *T*_4_ and CI was significantly elevated as compared to *T*_bsl_. In the CI-group SVI remained significantly lower as compared to *T*_bsl_. Mean arterial pressure and heart rate showed a similar pattern in both groups, but in the CI-group heart rate remained significantly higher by *T*_4_ as compared to *T*_bsl_, while it normalized in the SVI-group. Mean arterial pressure changed significantly in each group with a similar pattern without significant differences between the groups. There was less change in the CVP throughout the experiment, with a significant increase at *T*_3_ and *T*_4_ only in the SVI-group. Global end-diastolic volume decreased and then increased in both groups, but while it normalized by *T*_4_ in the SVI-group, it remained significantly lower in the CI-group as compared to the SVI-group and as compared to *T*_bsl_. Stroke volume variation and PPV also followed a similar pattern, and SVV normalized in the SVI-group but it remained significantly elevated in the CI-group, both as compared to *T*_bsl_ and between the groups at *T*_4_. Contractility, as indicated by dPmax values did not show any considerable change over time or between the groups.

### 3.2. Measures of Oxygen Debt

Oxygen delivery followed a similar pattern in both groups, but in the CI-group it remained significantly lower at *T*_4_ as compared to *T*_bsl_ (Table 3). In the SVI-group there was also a considerable drop by *T*_4_, although it was not significant. This can be explained by the significant and steady decrease in the hemoglobin levels in both groups. Oxygen consumption was more or less stable throughout the experiment, apart from a significant increase during the bleeding phase in both groups. Oxygen extraction changed accordingly with no major difference between the groups. Arterial pH, oxygen partial pressure, and oxygen saturation remained stable and within the normal range throughout.

Central venous oxygen saturation was in the normal range at *T*_bsl_ in both groups; then there was a significant drop after bleeding, which normalized in the SVI-group but remained significantly lower in the CI-group at *T*_4_ as compared to the SVI-group. The mean decrease in the CI-group from *T*_bsl_ to *T*_4_ was 15.1% and at *T*_4_ it was 8.8% lower as in the SVI-group. Central venous to arterial CO_2_-gap was normal at *T*_bsl_ in both groups. After bleeding it increased significantly and returned to normal in the SVI-group. In the CI-groups levels also decreased but remained elevated, although they did not reach statistical significance.

Lactate levels were slightly elevated at *T*_bsl_ in both groups, with significant increase in the SVI-group, which reduced by *T*_4_. In the CI-group significant changes could not be observed, and there was no significant difference between the groups either.

## 4. Discussion

In this study CI-based resuscitation resulted in residual hypovolemia compared to SVI-based fluid management as indicated by both macro-hemodynamic indices and measures of oxygen debt in a bleeding-resuscitation animal experiment.

### 4.1. Fluid Resuscitation

Fluid therapy is often regarded as the first line of support in most shock states and this holds especially true for acute bleeding. Fluid infusions directly increase intravascular volume and subsequently improve global and regional perfusion and oxygen delivery. However, this benefit can only occur in patients who are on the ascending limb of the Frank-Starling curve. In patients, who are regarded hypovolemic, only 50% respond to fluid, as defined by a 10–15% increase in stroke volume [11]. Although fluid resuscitation is a potentially lifesaving intervention large volumes can result in severe tissue edema and clinical signs of volume overload. These effects are mainly articulated in encapsulated organs, which have limited capacity to accommodate additional volume without compromising tissue perfusion. There is mounting evidence that both hypovolemia and fluid overload are associated with impaired organ function and increased risk of dying [2, 12, 13]. Therefore, adequate monitoring and defining appropriate resuscitation end points are of pivotal importance. However, according to recent large international surveys physicians apply monitoring and indicate fluid therapy based mainly on parameters, which are unable to predict fluid responsiveness. Several studies showed that mean arterial pressure and static markers of preload such as CVP, pulmonary capillary occlusion pressure have limited value in guiding fluid management; however more than 80% of physicians working in anesthesiology or in critical care still rely mainly on these parameters [14, 15]. Over the last 20 years there were 21 clinical trials published on perioperative goal-directed therapy [16]. In these studies hemodynamic goals showed a great variability. The most frequently used parameters to guide fluid management were CI, SV, SVV, PPV, CVP, MAP, echo-derived dynamic indices, pulmonary artery occlusion pressure, DO_2_, and oxygen extraction ratio. This clearly shows that universally accepted hemodynamic target by which fluid therapy should be tailored is missing.

It is important to note that recent milestones of multicenter clinical trials on fluid therapy [17–20] “neglected” this approach to some extent, and in these studies fluid administration was mainly based on the clinicians' “intuition” or inadequate indices rather than appropriate hemodynamic parameters of intravascular blood volume. Nevertheless, one of the most important messages of these large trials, which is also in accord with the results of recent surveys [14, 15], is that our everyday routine practice should be revised and may be harmful.

The physiological rationale of intravenous fluid administration to a patient is to increase SV, hence DO_2_, and also perfusion. In several studies CI was applied as therapeutic goal [21–24], although CO is the product of heart rate and SV; therefore compensatory mechanisms, such as tachycardia, may compensate residual hypovolemia. In the current experiment we found major differences between the SVI- and CI-guided groups. The latter received significantly less fluid in total and also required less fluid to replace every unit of lost blood. These results suggest that simply applying invasive hemodynamics as compared to our daily routine monitoring may not be sufficient, and depending on the parameter we chose to follow, subjects can still remain under- or overresuscitated.

### 4.2. SVI- versus CI-Guided Goal-Directed Resuscitation: Hemodynamics

During bleeding to restore homeostasis, the sympathetic nervous system becomes activated and releases epinephrine and norepinephrine. As a result, venous return will increase, while on the arterial side norepinephrine-caused vasoconstriction tries to maintain perfusion. Because of this sympathetic activation, heart rate and myocardial contractility will also increase. During resuscitation, our pivotal goal is to restore circulating blood volume by increasing SV to improve oxygen delivery. Recent clinical investigations [25, 26] showed positive effects of SV optimization, and there is frank evidence that PPV and SVV are well-established indicators of fluid responsiveness in mechanically ventilated subjects without cardiac arrhythmias [27]. Therefore in our experiment, SVV was the primary outcome variable as the closest to predict fluid responsiveness, hence hypovolemia. In both groups there was a significant increase after bleeding but values returned to baseline only in the SVI-group. In the CI-group neither dynamic (SVV/PPV) nor static indicators of preload (GEDI) normalized to their baseline values, indicating, that it was not the circulating blood volume, but heart rate compensated CO, which normalized, leaving residual hypovolemia unnoticed.

It is interesting to note that CVP changed to a lesser degree than any other hemodynamic parameter; hence our results provide further evidence of the limitations to CVP as a goal during fluid resuscitation, also described by others [7]. Although mean arterial pressure followed hemodynamic changes to some extent, there was no difference between the groups, indicating that for fine tuning hemodynamics, just as CVP, MAP also has limited value. This is due to the fact that MAP and CI do not correlate with each other [28].

However, “normalizing” global hemodynamics is one thing, but normalizing the balance between oxygen delivery and consumption is another. Therefore, once the macro-hemodynamic parameters look physiological, their effect on DO_2_/VO_2_ should also be assessed.

### 4.3. SVI- versus CI-Guided Goal-Directed Resuscitation: Oxygen Debt

As it has already been mentioned the primary goal of fluid resuscitation in hypovolemia is to maintain adequate oxygen delivery to the tissues. During bleeding, when oxygen demand/consumption is unchanged (in anesthetized subjects) or increased (in awake subjects), impaired oxygen delivery has to be accompanied by increased oxygen extraction ratio, which can be detected in the changes of S_cv_O_2_. Physiological mixed venous oxygen saturation ranges between 68% and 77%, and S_cv_O_2_ is considered to be 5% higher [29]. Indeed, in patients under general anesthesia the S_cv_O_2_ is often higher than 80%, which is due to the reduced oxygen demand and consumption; hence, higher values should be considered as “normal” [30, 31]. Furthermore, in our previous experiments, S_cv_O_2_ showed good correlation with oxygen extraction [32, 33]. Therefore, as interpretation of absolute values may prove difficult in different conditions evaluation of the changes of S_cv_O_2_ may be more helpful. In the current experiment we found that S_cv_O_2_ improved but remained significantly lower at the end of the experiment as compared to baseline values in both groups. This is most likely due to hemodilution, a feature also found in our previous study [9]. However, in the CI-group S_cv_O_2_ remained 15% lower as compared to baseline and more than 10% lower as in the SVI-group, indicating severe oxygen debt. Although interpreting S_cv_O_2_ may be difficult when there is problem with extraction typically seen in sepsis, there is international consensus that low levels should be an important warning sign to indicate inadequate DO_2_ to meet oxygen demands [34]. In our experiment in the CI-group, we measured lower DO_2_, S_cv_O_2_ and higher oxygen extraction ratio, indicating that animals resuscitated for CI remained in oxygen debt.

Several authors have reported increased dCO_2_ in different low flow states [35–38]. In hypoxemia caused anaerobic metabolism, hydrogen ions are generated by the hydrolysis of adenosine triphosphate to adenosine diphosphate, and by the increased production of lactic acid [36]. These hydrogen ions are buffered by bicarbonate present in the cells, and this process will generate CO_2_ production [37]. Arterial PaCO_2_ is dependent on pulmonary gas exchange, and central venous PvCO_2_ is dependent on the capability of blood flow to wash out carbon dioxide from the tissues. The Fick principle adapted to carbon dioxide demonstrates the inverse relationship between CO and dCO_2_ [39]. Thus, it has been postulated that increased dCO_2_ reflects decreased flow. In the current experiment dCO_2_ followed the same pattern, what we observed previously, and returned to the baseline value at the end of resuscitation in the SVI-group. In the CI-group, it remained elevated, above the physiological value of 6 mmHg, but this difference did not reach statistical significance. Nevertheless, this tendency gives further evidence that these animals were underresuscitated.

Lactate, the product of anaerobic metabolism, is often referred to as one of the main biochemical targets to be treated during resuscitation [40]. In our experiment, levels were slightly elevated at baseline, possibly due to the relatively long set-up time of the experiment, and there was an increase and then decrease during interventions, but these changes were not as dramatic as one may expect. However, it is important to note, that this experimental model is similar to a “moderate” bleeding event, and animals were resuscitated within a relatively short period of time. Due to the physiologic relationship between DO_2_ and VO_2_, namely, due to compensatory mechanisms when there is a drop in DO_2_, up to a certain point VO_2_ remains stable, in other words independent from DO_2_. Therefore, although the VO_2_/DO_2_ ratio is increasing, but it does not cause and mean cellular hypoxia, hence aerobic metabolism is not disturbed. To conclude, animals during this experiment were heading towards shock; they were in oxygen debt but remained on the flat part of the VO_2_/DO_2_ curve, not reaching cellular hypoxia and shock, meaning the steep part of the curve. This is also supported by the arterial pH, which remained normal throughout in both groups. In general, this is the rationale and advantage of measuring S_cv_O_2_, and for similar reasons SVV or PPV, because we are “one step ahead” of cellular hypoxia and circulatory shock.

### 4.4. Limitations

One of the limitations of this experiment is that we could not provide data on microcirculation and regional blood flow, which would be interesting to see. Furthermore, these results can only partially be extrapolated for the real clinical settings. Reducing the SVI by 50% is a strictly controlled scenario, rarely happening in the everyday practice. The observation period at the end of the experiment was also short; therefore, long-term effects of SVI or CI-based fluid resuscitation could not be assessed. Another limitation of the model is that bleeding was relatively fast, causing a sympathetic burst, which is a reality in trauma and when major bleeding occurs on the wards, but in the operating room intravascular volume loss and bleeding caused hypovolemia usually occurs over a longer period of time.

## 5. Conclusion

In this experiment we have shown that SVI-based goal-directed resuscitation of a bleeding subject seems superior to CI-guided resuscitation as indicated by both hemodynamic parameters and measures of oxygen debt returning to baseline in the SVI-group, which was incomplete in the CI-group. However, we would like to emphasize that treating one single parameter during resuscitation is not warranted. It is not one single parameter, but the “hemodynamic puzzle” what we have to solve [41]. Therefore, it is necessary to put hemodynamic variables and measures of VO_2_/DO_2_ into context in a way that once macro-hemodynamic parameters are “normalized,” adequacy of treatment has to be checked by measures of oxygen debt. Measuring SVV or PPV and simple blood gas driven variables such as S_cv_O_2_ and dCO_2_ are valuable tools to solve this puzzle as quickly as possible.

## Figures and Tables

**Table 1:** Demographics and fluid therapy.

	SVI-group (*n* = 14)	CI-group (*n* = 12)	*p*
Weight (kg)	29.00 ± 5.36	27.54 ± 5.46	0.606
BSA (m^2^)	0.98 ± 0.09	0.93 ± 0.91	0.390
Shed blood (mL)	485 ± 91	479 ± 101	0.859
Shed blood (mL/m^2^)	492 ± 59	508 ± 101	0.719
Total amount of the replaced fluid (mL)	1965 [1584–2165]	900 [850–1780]	0.020^*∗*^
Required fluid (mL)/unit blood loss (10 mL)	40 ± 12	25 ± 12	0.027^*∗*^

SVI (stroke volume index), SVI-group; CI (cardiac index), CI-group. Data are presented as mean ± standard deviation or median [interquartile range] as appropriate. ^*∗*^ *p* < 0.05.

**Table 2:** Hemodynamic parameters during hemorrhage and fluid resuscitation.

	Group	*T*_bsl_	*T*_0_	*T*_1_	*T*_2_	*T*_3_	*T*_4_
Stroke volume index (mL/m^2^)	SVI	27.5 ± 5.4	13.8 ± 2.6^*∗*^	16.5 ± 2.8^*∗*^	19.5 ± 3.7^*∗*#^	23.6 ± 5.1^#^	28.0 ± 5.0^#^
	CI	31.4 ± 4.7	14.4 ± 9.0^*∗*^	18.1 ± 3.6^*∗*^	19.2 ± 3.6^*∗*^	23.2 ± 1.3^*∗*#^	23.8 ± 5.9^*∗*#@^

Cardiac index (L/min/m^2^)	SVI	2.6 ± 0.3	1.8 ± 0.3^*∗*^	2.1 ± 0.4^*∗*^	2.4 ± 0.3^#^	2.7 ± 0.4^#^	2.9 ± 0.4^*∗*#^
	CI	2.8 ± 0.3	1.7 ± 0.5^*∗*^	2.1 ± 0.3^*∗*^	2.4 ± 0.2^#^	2.6 ± 0.4^#^	2.7 ± 0.3^#^

Mean arterial pressure (mmHg)	SVI	116 ± 17	72 ± 17^*∗*^	75 ± 19^*∗*^	78 ± 18^*∗*^	86 ± 17^*∗*^	92 ± 16^*∗*#^
	CI	124 ± 12	75 ± 22^*∗*^	77 ± 18^*∗*^	80 ± 81^*∗*^	86 ± 22^*∗*^	96 ± 20^*∗*#^

Heart rate (beats/min)	SVI	95 ± 13	133 ± 22^*∗*^	130 ± 29^*∗*^	121 ± 21^*∗*^	111 ± 18^#^	101 ± 12^#^
	CI	89 ± 11	139 ± 37^*∗*^	131 ± 13^*∗*^	127 ± 28^*∗*^	121 ± 24^*∗*^	117 ± 35^*∗*^

Central venous pressure (mmHg)	SVI	5.9 ± 1.0	4.8 ± 0.7	5.5 ± 1.9	5.6 ± 1.4	6.1 ± 1.2^#^	6.2 ± 1.3^#^
	CI	6.0 ± 0.6	4.7 ± 0.8	5.3 ± 0.6	5.6 ± 0.5	6.2 ± 1.5	6.5 ± 0.7

Global end-diastolic volume (mL/m^2^)	SVI	308 ± 56	237 ± 61^*∗*^	243 ± 59^*∗*^	251 ± 46^*∗*^	282 ± 58^#^	298 ± 53^#^
	CI	312 ± 33	191 ± 56^*∗*@^	204 ± 32^*∗*^	211 ± 27^*∗*^	243 ± 32^*∗*#^	247 ± 32^*∗*#@^

Stroke volume variation (%)	SVI	14.7 ± 4.7	22.1 ± 5.5^*∗*^	22.2 ± 4.9^*∗*^	18.5 ± 4.6	16.7 ± 5.2^#^	12.1 ± 3.6^#^
	CI	11.5 ± 5.3	18.6 ± 5.2^*∗*^	18.7 ± 3.7^*∗*^	21.3 ± 4.8	19.3 ± 4.1^*∗*^	17.4 ± 7.6^*∗*@^

Pulse pressure variation (%)	SVI	14.2 ± 5.3	24.6 ± 6.9^*∗*^	23.3 ± 6.7^*∗*^	19.0 ± 5.8^#^	16.7 ± 5.2^#^	13.1 ± 4.1^#^
	CI	12.2 ± 3.1	25.2 ± 6.7^*∗*^	22.8 ± 5.4^*∗*^	19.8 ± 4.5^#^	17.4 ± 5.8^#^	16.3 ± 6.7^#^

Systemic vascular resistance index (dyn×s/cm^5^/m^2^)	SVI	3261 ± 942	3100 ± 873	2677 ± 734	2442 ± 698^*∗*#^	2410 ± 466^*∗*#^	2336 ± 475^*∗*#^
	CI	3507 ± 597	3191 ± 709	2767 ± 630^*∗*^	2652 ± 240^*∗*^	2508 ± 565^*∗*^	2481 ± 495^*∗*#^

EVLWI (mL/kg)	SVI	10.1 ± 1.9	10.0 ± 2.2	9.9 ± 1.9	9.0 ± 1.5	9.3 ± 1.6	9.8 ± 1.7
	CI	7.4 ± 1.2^@^	7.2 ± 0.9^@^	7.2 ± 1.0^@^	7.4 ± 1.0	7.5 ± 0.9^@^	8.2 ± 1.0^@^

dPmax (mmHg/s)	SVI	561 ± 226	560 ± 344	653 ± 404	682 ± 390	987 ± 269	674 ± 236
	CI	585 ± 87	595 ± 206	579 ± 95	597 ± 137	551 ± 105	639 ± 154

SVI (stroke volume index), SVI-group; CI (cardiac index), CI-group. Data are presented as mean ± standard deviation.

^*∗*^ *p* < 0.05 significantly different from *T*_bsl_.

^#^ *p* < 0.05 significantly different from *T*_0_.

^@^ *p* < 0.05 significantly different between groups.

**Table 3:** Blood gas parameters during hemorrhage and fluid resuscitation.

	Group	*T*_bsl_	*T*_0_	*T*_1_	*T*_2_	*T*_3_	*T*_4_
Oxygen delivery index (mL/min/m^2^)	SVI	417 ± 64	250 ± 100^*∗*^	275 ± 71^*∗*^	291 ± 55^*∗*^	318 ± 55^*∗*^	337 ± 81^#^
	CI	410 ± 54	271 ± 62^*∗*^	297 ± 85^*∗*^	282 ± 47^*∗*^	278 ± 52^*∗*^	311 ± 61^*∗*^

Oxygen consumption (index mL/min/m^2^)	SVI	82 ± 27	118 ± 63^*∗*^	111 ± 19	102 ± 24	98 ± 24	94 ± 23
	CI	71 ± 43	115 ± 48^*∗*^	111 ± 29	108 ± 21	103 ± 18	99 ± 13

Oxygen extraction (VO_2_/DO_2_)	SVI	0.20 ± 0.06	0.40 ± 0.11^*∗*^	0.36 ± 0.06^*∗*^	0.33 ± 0.11^*∗*^	0.29 ± 0.09^*∗*#^	0.27 ± 0.13^#^
	CI	0.17 ± 0.09	0.40 ± 0.18^*∗*^	0.38 ± 0.09^*∗*^	0.36 ± 0.08^*∗*^	0.34 ± 0.14^*∗*^	0.33 ± 0.11^*∗*^

Arterial pH	SVI	7.48 ± 0.04	7.46 ± 0.07	7.44 ± 0.06	7.41 ± 0.11	7.45 ± 0.04	7.46 ± 0.04
	CI	7.44 ± 0.04	7.43 ± 0.06	7.42 ± 0.05	7.47 ± 0.03	7.42 ± 0.05	7.45 ± 0.05

Partial pressure of oxygen in arterial blood (mmHg)	SVI	94.5 ± 26.5	94.9 ± 27.8	90.1 ± 20.2	94.9 ± 27.1	93.1 ± 27.1	95.5 ± 30.1
	CI	88.3 ± 28.8	89.8 ± 28.8	97.6 ± 30.2	89.2 ± 22.5	93.8 ± 32.6	88.2 ± 27.6

Arterial oxygen saturation (%)	SVI	97.3 ± 1.5	96.7 ± 2.1	96.3 ± 2.0	97.0 ± 1.5	97.0 ± 1.7	96.9 ± 1.8
	CI	95.4 ± 3.6	95.3 ± 5.0	96.1 ± 4.2	98.6 ± 1.5	95.6 ± 4.8	96.0 ± 3.2

Central venous oxygen saturation (%)	SVI	77.4 ± 6.6	57.5 ± 10.8^*∗*^	60.9 ± 4.8^*∗*^	64.3 ± 9.2^*∗*^	68.4 ± 8.6^*∗*#^	72.9 ± 7.5
	CI	79.2 ± 8.1	56.7 ± 17.0^*∗*^	58.5 ± 10.6	59.7 ± 8.0	63.0 ± 14.7	64.1 ± 11.6^#@^

Venous to arterial carbon dioxide gap (mmHg)	SVI	5.7 ± 2.4	10.1 ± 2.6^*∗*^	8.9 ± 1.7	7.5 ± 2.4	7.2 ± 2.7	5.3 ± 2.3^#^
	CI	4.0 ± 3.1	9.9 ± 6.0^*∗*^	8.8 ± 2.4^*∗*^	8.5 ± 3.0	8.1 ± 3.1	7.6 ± 4.3

Lactate (mmol/L)	SVI	2.54 ± 1.01	3.97 ± 1.80^*∗*^	4.72 ± 2.29^*∗*^	4.37 ± 2.37^*∗*^	3.90 ± 2.25^*∗*^	3.26 ± 1.95
	CI	3.32 ± 1.26	4.49 ± 1.83	4.50 ± 2.40	4.32 ± 0.69	4.05 ± 2.52	3.77 ± 2.32

Hemoglobin (g/dL)	SVI	11.6 ± 1.5	10.7 ± 1.5	10.4 ± 1.46^*∗*^	9.4 ± 1.2^*∗*#^	8.3 ± 1.5^*∗*#^	8.1 ± 0.9^*∗*#^
	CI	11.2 ± 0.7	10.4 ± 1.2	9.5 ± 1.2^*∗*^	9.2 ± 0.9^*∗*#^	8.4 ± 0.6^*∗*#^	8.2 ± 1.5^*∗*#^

SVI (stroke volume index), SVI-group; CI (cardiac index), CI-group. Data are presented as mean ± standard deviation.

^*∗*^ *p* < 0.05 significantly different from *T*_bsl_.

^#^ *p* < 0.05 significantly different from *T*_0_.

^@^ *p* < 0.05 significantly different between groups.
